# A Low-Cost and Efficient Indoor Fusion Localization Method

**DOI:** 10.3390/s22155505

**Published:** 2022-07-23

**Authors:** Suqing Yan, Chunping Wu, Honggao Deng, Xiaonan Luo, Yuanfa Ji, Jianming Xiao

**Affiliations:** 1 Guangxi Key Laboratory of Precision Navigation Technology and Application, Guilin University of Electronic Technology, Guilin 541004, China; yansuqing@guet.edu.cn (S.Y.); jiyuanfa@163.com (Y.J.); 2 School of Information and Communication, Guilin University of Electronic Technology, Guilin 541004, China; wuchunping1@163.com; 3 Guangxi Key Laboratory of Image and Graphic Intelligent Processing, Guilin University of Electronic Technology, Guilin 541004, China; luoxn@guet.edu.cn; 4 National & Local Joint Engineering Research Center of Satellite Navigation Positioning and Location Service, Guilin 541004, China; 5 Department of Science and Engineering, Guilin University, Guilin 541004, China; xjm_netease@163.com

**Keywords:** indoor localization, step length estimation, acoustic signal, improved pedestrian dead reckon

## Abstract

Accurate indoor location information has considerable social and economic value in applications, such as pedestrian heatmapping and indoor navigation. Ultrasonic-based approaches have received significant attention mainly since they have advantages in terms of positioning with temporal correlation. However, it is a great challenge to gain accurate indoor localization due to complex indoor environments such as non-uniform indoor facilities. To address this problem, we propose a fusion localization method in the indoor environment that integrates the localization information of inertial sensors and acoustic signals. Meanwhile, the threshold scheme is used to eliminate outliers during the positioning process. In this paper, the estimated location is fused by the adaptive distance weight for the time difference of arrival (TDOA) estimation and improved pedestrian dead reckoning (PDR) estimation. Three experimental scenes have been developed. The experimental results demonstrate that the proposed method has higher localization accuracy in determining the pedestrian location than the state-of-the-art methods. It resolves the problem of outliers in indoor acoustic signal localization and cumulative errors in inertial sensors. The proposed method achieves better performance in the trade-off between localization accuracy and low cost.

## 1. Introduction

Location-based services (LBS) emerged under the promotion of technical development and social demands and have become a very popular research topic in recent years. According to statistics, 80% of people’s activities are completed in an indoor environment. The demands of indoor localization for indoor navigation services, and material positioning have been growing and have great social and commercial value and broad application prospects.

Researchers have conducted a series of related works on indoor localization technologies (e.g., WIFI fingerprints [1], geomagnetic [2] and ultrasonic [3] techniques). Of all of these technologies, the ultrasonic-based localization method is calculated using geometric distance. It has high localization accuracy for low propagation speeds compared with radio signals. Furthermore, ultrasonic-based localization systems do not require the deployment of extra devices due to their compatibility with smartphones. Refs. [4,5] used a time difference of arrival (TDOA) measurement for ultrasonic and radio frequency signals to measure the distance between two locations. Afterward, the location estimation was calculated using the trilateral method. Although indoor localization based on acoustic signals can achieve high-precision localization, it still cannot solve the errors caused by the indoor environments and building structures. Different ambient sound signals need to be specifically set according to the environment. The heterogeneity of buildings still hinders the universal application of acoustic signal localization.

In indoor localization based on PDR, data are collected by inertial sensors, which are not affected by the surrounding environments. At present, all smartphones and tablets on the market have been installed with the integrated inertial measurement unit (IMU), which solves the hardware problem of using this technology for localization. Refs. [6,7] describes the autocorrelation analysis method for step detection, which has high accuracy but poor adaptability to motion changes. The zero crossing detection method was used for step detection in [8]; it is easy to implement, but not suitable for real-time research. In [9,10], the peak detection method was used for step detection, and it has less resource consumption and good real-time performance. This method has the advantages of low cost and high autonomy without extra infrastructures. However, accumulated localization errors over time cause low localization accuracy.

In summary, it is difficult to balance cost, accuracy, and stability. Ultrasonic waves are susceptible to the surrounding environment (mirror reflection, limited beam angle, walls, shadows, etc.), and anomalies occur in the TDOA estimation, which can lead to large localization errors. Due to inertia, cumulative errors in PDR estimation hinder extensive application in realistic scenes. Single-mode indoor localization limits the application scenes in the existing localization technologies. In this paper, a multimodal fusion localization algorithm is proposed. Using this method, the localization accuracy is improved and the pervasiveness of location application is enhanced in an indoor scene. However, there are two major challenges in realizing such a fusion localization system.

- How can one balance low cost and high localization? In the popularization of applications, we often hope to achieve low cost and high precision to meet the requirements of localization. However, high precision often requires high-cost infrastructures. Therefore, the trade-off between cost and accuracy is the key to indoor localization.
- How can one fuse the acoustic and inertia localization? Different localization results vary with different methods. The contribution of each localization result is not uniform in the localization process. Therefore, determining the importance of each method and fusing the multimodal method remains a challenge.

To address the above challenges, we propose an ultrasonic-based fusion localization system. The system can achieve higher precision indoor localization with low cost and retain the advantages of the acoustic signals and PDR localization while overcoming the effects of acoustic localization occlusion and the PDR cumulative error. The main contributions of this paper are as follows:

- **An improved PDR method:** Based on the compatibility of mobile phones with ultrasonic signals and inertial sensor positioning, a fusion localization method is proposed to solve the contradiction between cost and accuracy. To gain high location estimation, we propose an improved PDR method. The experimental results show that our method has better localization performance than the traditional PDR method. Afterward, we propose a threshold method to eliminate the anomalies, and the localization accuracy is improved.
- **An adaptive weight based on the distance fusion scheme:** To balance the contribution of the localization results, we propose an adaptive weight based on a distance scheme that estimates the weight value for acoustic and improved PDR localization. Based on the weight values, we fuse the acoustic estimation and improved inertial estimation to achieve accurate pedestrian location.

The organization of the rest of the paper is as follows: in Section 2, related work is presented, followed by the workflow of our localization system in Section 3. We introduce the indoor localization system with sound signals, PDR, and fused localization algorithms in Section 4. The illustrative experimental results are provided in Section 5. Section 6 summarizes the research results presented in this paper.

Notations shows the major symbols used in this article.

## 2. Related Works

Indoor localization has been extensively studied for decades. Scholars have adopted various signals to develop related works, including ultra-wideband (UWB) [11,12,13], WIFI [14,15,16], Radio Frequency Identification (RFID) [17,18], Bluetooth [19,20], Geomagnetic sequence [21,22] and Ultrasonic signals [3,23]. Despite the accuracy in special trial sites, they have a few practical limitations that hinder their wide deployment. UWB-based and RFID-based systems need to install special infrastructure, which has high costs. Infrared-based localization technology is often blocked by indoor building structures and has no pervasiveness due to complex indoor environments. WIFI signals, Bluetooth signals and geomagnetic sequences need to construct a fingerprint database beforehand and are updated from time to time to adapt to surrounding environment changes. More human resources are needed.

Localization based on ultrasonic signals has recently attracted much attention, mainly since it is compatible with mobile phones, deployable without infrastructure support, and has high localization accuracy. Therefore, researchers have begun to study indoor localization with ultrasonic signals. The typical localization system based on ultrasonic signals [24,25] uses the TDOA to estimate the pedestrian location. Shuangshuang Li [26] et al. proposed a TDOA-based localization algorithm for underwater acoustic sensor networks. In this network, the maximum likelihood (ML) ratio criterion was adopted to reduce acoustic localization errors. Ref. [27] presented an acoustic localization algorithm based on a decadal stereo array. Gergely Vakulya [28] et al. proposed an adaptive consistency-function-based solution localization algorithm for cooperative and consistent errors. Suresh Manickam [29] et al. proposed a multisource distributed localization algorithm. Hu et al. proposed a TDOA localization method based on improved the TPSN and KF [30], and it improved the accuracy due to time synchronization. Yu-Ting Wang et al. proposed a zero-configuration indoor localization solution using asynchronous acoustic beacons [31]. Based on the clock synchronization problem in the TDOA real-time localization system, Ge Yan et al. proposed a cross-check synchronization method [32] and the soft clock synchronization design of the TDOA positioning system on the DW1000 module. The method contains the communication protocol of the master base station with slave base stations and user tags and arranges the communication time slots between modules to solve the problem of mutual interference of communication signals. Liu [33] first proposed the positioning system GuoGuo using a smartphone as a positioning terminal and achieved a positioning accuracy of 6–25 cm using a pseudo-random code acoustic signal in the 15 kHz to 20 kHz frequency band. Patrick Lazik [34] et al. achieved a localization accuracy of 10 cm using chirp acoustic signals.

Inertial measurement technology does not rely on additional equipment for auxiliary localization. It can be located in all weather and terrain. Lei Cheng [35] et al. proposed a fusion method that adopted the Kalman filter to fuse binocular vision and an inertial navigation system (INS). Jishi Cui [36] et al. proposed an indoor localization system in which an improved Zee method and regularized particle filters were used to improve the cumulative error of the PDR algorithm. Rohan Kumar Yadav [37] et al. proposed an indoor localization method based on BLE beacons and IMUs to enhance accuracy.

Localization based on acoustic and inertial sensors has made remarkable achievements. However, environmental error is still not well eliminated. Therefore, the single acoustic localization method still cannot meet the practical requirements of cost and accuracy. Combining acoustic localization with IMU auxiliary localization is a good scheme for environmental effects, and this strategy not only achieves high accuracy but also has relatively low equipment cost. The multimodal fusion system that we propose adopts an adaptive distance weighting approach to estimate the location.

## 3. System Workflow

**Figure 1:**
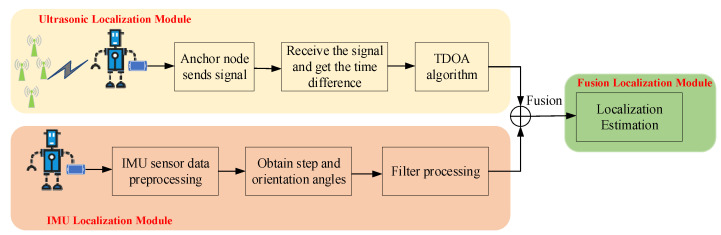
Overall scheme of the fusion localization system.

In this section, we overview the workflow of the fusion localization method in Figure 1, which includes three parts: the TDOA estimation-based ultrasonic signal, the estimation based IMU and the fusion localization estimation.

In this workflow, the first stage involves the collection of ultrasonic and inertial sensor signals. The volunteer holds smartphone in the heading direction at constant speed. The ultrasonic and inertial sensor signals are collected automatically by the client applications installed on the smartphone, and sent to the server. In the server ultrasonic signals are extracted and preprocessed, and the TDOA estimation module determines the target location. In localization based on IMU, the acceleration, gyroscope, magnetometer and heading angles are extracted from the server and are preprocessed. According to the IMU data, we obtain the target location using the improved PDR method. Finally, accuracy localization estimation is achieved by fusing the TDOA estimation and IMU estimation.

## 4. Fusion Localization Based on Ultrasonic and Inertial Signals

We illustrate the design of the fusion localization method and provide an overview of the proposed fusion system in Section 4.1. Afterward, Section 4.2 illustrates data extraction and preprocessing. Section 4.3 introduces the estimation based on acoustic and IMU signals, and Section 4.4 illustrates the adaptive weight value generation. Finally, the fusion localization estimation is shown in Section 4.5.

### 4.1. Overview

**Figure 2:**
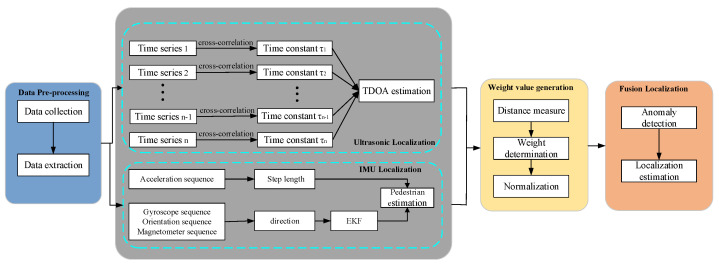
Detailed technical implementation of the proposed fuse localization system.

In this section, we introduce the multimodal fusion localization system. The detailed technical implementation structure is presented in Figure 2. First, we extract the ultrasonic signal, acceleration, magnetometer and gyroscope sequences from the client. Afterward, we adopt the TDOA algorithm to determine the target with ultrasonic signals. For the acceleration, magnetometer and gyroscope sequences, the improved PDR algorithm, which localizes the target, is presented. To identify the importance, we generate weight values for the two methods. Finally, we fuse the two locations with adaptive weight values to obtain the final location for the target.

More specifically, our system includes the following components:

*Data extraction and preprocessing:* In this part, we collect the data using the application installed on the smartphone and send data to servers. The ultrasonic signals and inertial data from the IMU are extracted from the received data.

*Target coarse determination:* In this module, the location of the target is perceived using ultrasonic signals and inertial data. The TDOA algorithm is adopted to calculate the target location with acoustic signals. For the inertial data, the improved PDR is presented to estimate the target.

*Adaptive weight value generation:* The different models in each location have different importance. After finishing the target coarse determination, we propose an adaptive weight value generation mechanism based on the localization of the current time and former time.

*Fusion localization:* Based on the weight value, we design a fusion localization estimation method for ultrasonic and inertial sequences. 

### 4.2. Data Extraction and Preprocessing

A chirp signal is a widely used pulse compression signal, and it is a typical non-smooth signal with good autocorrelation characteristics. It can still be detected by correlation operations even with severe signal fading. In this paper, the chirp signal is used as the ultrasonic signal, which is defined as follows:

(1) $$s\left(t\right)=e^{j2\pi \left(f_{0}t+\frac{1}{2}k_{0}t^{2}\right)},t\in \left[0,T\right]$$

where $f_{0}$ is the initial frequency of the chirp signal, $k_{0}$ is the modulation rate, and T is the duration time.

**Figure 3:**
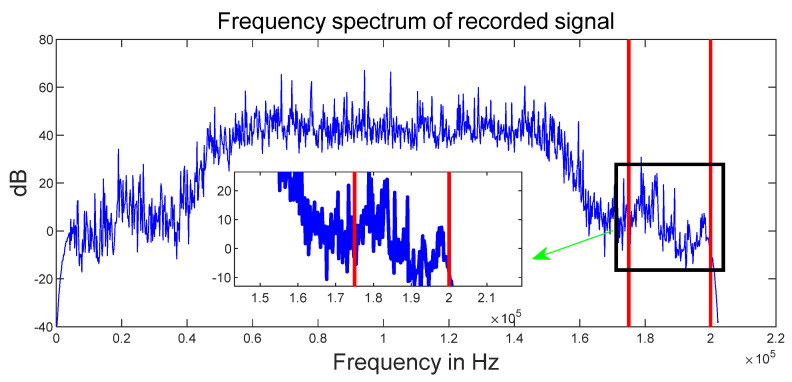
Spectrum analysis of in vivo Y85a transmit and MEIZU 16 s Pro receive signals.

Signals with different frequency ranges have various transmission characteristics in indoor environments. To validate this assertion, we sample the acoustic signal with an in vivo Y85a smartphone, in which the frequency range is 0−25 kHz, and the acoustic source is HUAWEI Nova 4 devices. Figure 3 shows the spectrum of the received signals at the trial site. The spectrums of acoustic signals are relatively stable below 15 kHz and the range of 17−20 kHz, and a sharp drop between 15 kHz and 17 kHz. Therefore, considering the requirements of indoor localization, we choose chirp signals with a frequency range of 17−19 kHz.

We deployed multiple acoustic anchors with a pair of microphones and speakers at fixed locations. The microphone and speaker at the anchor point are spatially separated. Each anchor message consists of a preamble, an identifier (*id*), a sequence number (*seqno*.) and local timestamp (*ts*). The anchor node decodes the message from the anchors including itself. The target devices passively listen to the acoustic anchors and save the *id*, *seqno*. and timestamps from the received beacon messages. The server program calculates the location of the mobile device with the related information.

We developed the corresponding data acquisition application, which is based on the Android platform, for smartphones. While collecting ultrasonic signals, the target also collects acceleration, gyroscope and direction data. The various sensor data collected by the phone are stored in *.txt format.

### 4.3. Target Estimation from Acoustic Signal and IMU

In this section, we illustrate the target coarse estimation, which includes the ultrasonic and inertial sequences.

#### 4.3.1. Time Difference of Arrival Estimation

**Figure 4:**
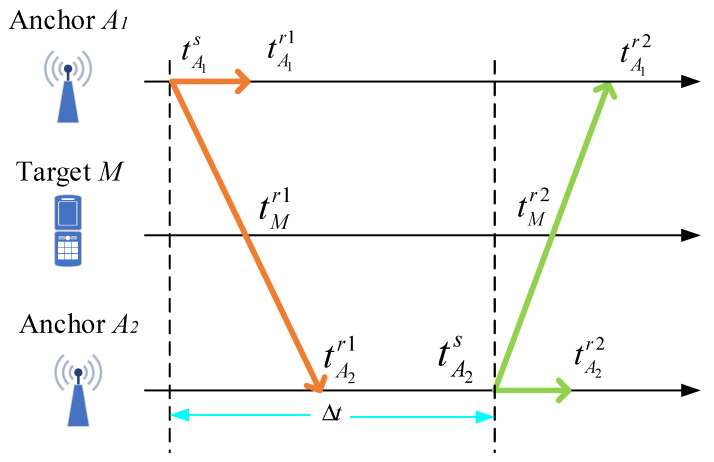
Diagram of message transmission and reception between two anchors and one target.

We detail the TDOA estimation for the ultrasonic localization in Figure 4. Let the system comprise anchors *A_1_* and *A_2_* and target *M*.

Suppose at time $t_{A_{1}}^{s}$ anchor *A_1_* transmits a message that arrives at anchor *A_1_*, *A_2_* and *M* at $t_{A_{1}}^{r1},\;t_{A_{2}}^{r1}$ and $t_{M}^{r1}$, respectively. At time $t_{A_{2}}^{r1}$, anchor *A_2_* sends a message, which is received at times $t_{A_{1}}^{r2},\;t_{A_{2}}^{r2}$ and $t_{M}^{r2}$, respectively. Here, it is unknown for $t_{A_{1}}^{s}$ and $t_{A_{2}}^{s}$.

Consider Δt to be the transmission interval between anchors *A_1_* and *A_2_* in a common reference time and $d_{A_{1}A_{1}}$ and $d_{A_{2}A_{2}}$ to be the distances from anchor *A_1_*’s speaker to the microphone and anchor *A_2_*’s speaker to the microphone, respectively. We use $d_{A_{i}A_{j}}$ to express the distance from anchor *A_i_*’s speaker to anchor *A_j_*’s microphone. Let *c* be the speed of acoustic propagation in the air.

We can obtain the timestamps *t_1_* and *t_2_* at which anchor *A_2_* sends messages from anchors *A_1_* and *A_2_*

(2) $$t_{1}=\left(t_{A_{1}}^{r2}-\frac{d_{A_{1}A_{2}}}{c}\right)$$

(3) $$t_{2}=\left(t_{A_{2}}^{r2}-\frac{d_{A_{2}A_{2}}}{c}\right)$$

The timestamps *t_3_* and *t_4_* at which anchor *A_1_* sends messages from anchors *A_1_* and *A_2_* are obtained separately

(4) $$t_{3}=\left(t_{A_{1}}^{r1}-\frac{d_{A_{1}A_{1}}}{c}\right)$$

(5) $$t_{4}=\left(t_{A_{2}}^{r1}-\frac{d_{A_{1}A_{2}}}{c}\right)$$

Therefore, the transmission interval Δt can be expressed as follows:

(6) $$\Delta t=t_{1}+t_{2}-t_{3}-t_{4}/2$$

Then, it can be further simplified as

(7) $$\Delta t=\frac{\left(t_{A_{2}}^{r2}-t_{A_{2}}^{r1}\right)+\left(t_{A_{1}}^{r2}-t_{A_{1}}^{r1}\right)}{2}$$

After determining the transmission interval Δt, the TDOA from anchors *A_1_* and *A_2_* on target *M* can be calculated. The time calculations are all based on the same device clock system for time subtraction. Therefore, the distances difference from anchors *A_1_* and *A_2_* to target *M* can be computed as

(8) $$d_{A_{1}A_{2}}=\left(t_{M}^{r2}-t_{M}^{r1}-\Delta t\right)*c$$

where *c* is the speed of acoustic signal propagation in the atmosphere.

**Figure 5:**
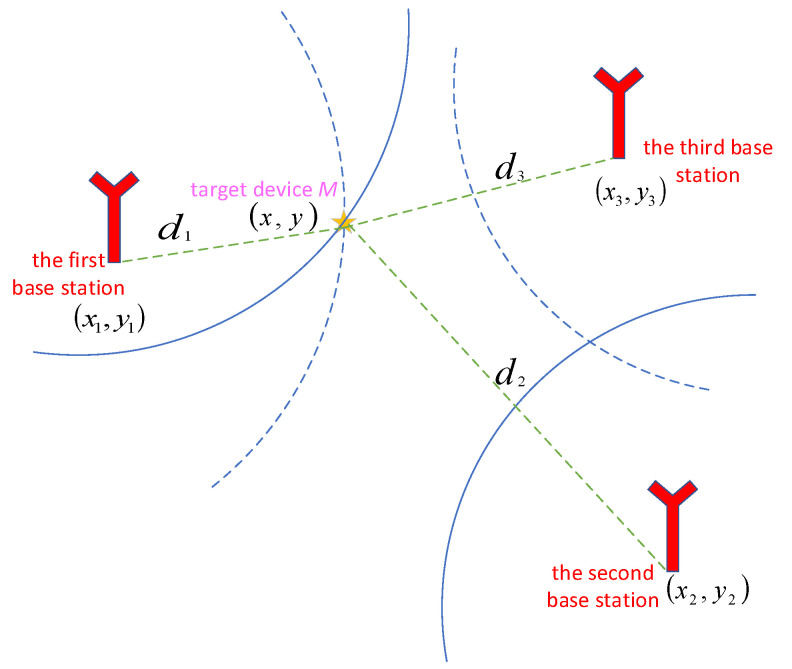
TDOA hyperbolic model.

At least three anchors are needed to achieve localization in the location estimation. The TDOA hyperbolic model [38] is shown in Figure 5. We assume the target location M(x,y) and anchor locations $A_{1}\left(x_{1},y_{1}\right)$, $A_{2}\left(x_{2},y_{2}\right),$ $A_{3}\left(x_{3},y_{3}\right)$. $d_{ij}$ is the distance difference from anchors *A_i_* and *A_j_* on Target *M*. The location can be achieved by using Equation (9).

(9) $$\{\begin{array}{c} {\left\|(A_{2}-M)\right\|}_{2}-\|{\left(A_{1}-M)\right\|}_{2}=d_{21}\\ {\left\|(A_{3}-M)\right\|}_{2}-{\left\|(A_{1}-M)\right\|}_{2}=d_{31} \end{array}$$

Suppose *A_i_*’s location is $\left(X_{i},Y_{i}\right)$. Then, the distance between the target *M* and anchor *A_i_* is

(10) $$d_{i}=\sqrt{{\left(X_{i}-x\right)}^{2}+{\left(Y_{i}-y\right)}^{2}}$$

(11) $$d_{i}{}^{2}={\left(X_{i}-x\right)}^{2}+{\left(Y_{i}-y\right)}^{2}=P_{i}-2X_{i}x-2Y_{i}y+x^{2}+y^{2}$$

where $P_{i}=X_{i}{}^{2}+Y_{i}{}^{2}$.

Let $d_{i,1}$ denote the distance difference from anchors *A_i_* and *A_1_* on Target *M* then

(12) $$d_{i,1}=d_{i}-d_{1}$$

We can obtain

(13) $$d_{i}{}^{2}={\left(d_{i,1}+d_{1}\right)}^{2}$$

Equation (13) can be expanded and expressed as

(14) $$d_{i,1}{}^{2}+2d_{i,1}d_{1}+d_{1}{}^{2}=P_{i}-2X_{i}x-2Y_{i}y+x^{2}+y^{2}$$

When *i* = 1, Equation (11) simplifies to

(15) $$d_{1}{}^{2}=P_{1}-2X_{1}x-2Y_{1}y+x^{2}+y^{2}$$

Equation (14) is subtracted from Equation (15) to obtain

(16) $$d_{i,1}{}^{2}+2d_{i,1}d_{1}=P_{i}-2X_{i,1}x-2Y_{i,1}y-P_{1}$$

where $X_{i,1}=X_{i}-X_{1}$,$\;Y_{i,1}=Y_{i}-Y_{1}$.

From Equation (16), we obtain

(17) $$d_{i,1}{}^{2}-P_{i}+P_{1}=-2d_{i,1}d_{1}-2X_{i,1}x-2Y_{i,1}y$$

Then, we can obtain the matrix

(18) Bq=b 

where

(19) $$B=\left(-2\right)\left[\begin{array}{ccc} d_{21} & X_{21} & Y_{21}\\ d_{31} & X_{31} & Y_{31}\\ d_{41} & X_{41} & Y_{41} \end{array}\right]$$

(20) $$b=\left[\begin{array}{c} d_{21}{}^{2}-P_{2}+P_{1}\\ d_{31}{}^{2}-P_{3}+P_{1}\\ d_{41}{}^{2}-P_{4}+P_{1} \end{array}\right]$$

(21) $$q=\left[\begin{array}{c} d_{1}\\ x\\ y \end{array}\right]$$

The location can be computed as

(22) $$q={\left(B^{T}B\right)}^{-1}b\;$$

#### 4.3.2. Improved Pedestrian Dead Reckon Estimation

In this part, we elaborate on the improved pedestrian dead reckoning algorithm, which includes step length estimation, heading direction, and dead reckon estimation.

- Step length estimation

While walking, the acceleration of the pedestrian changes approximately periodically. The alternating transformation of the peak and valley of the acceleration wave once is equivalent to one step, so peak detection can be used to estimate the step frequency and step length. The detection process is mainly divided into the following three parts:

(1) Peak detection:

If the acceleration a(t) at time *t* is greater than the acceleration a(t−1) at time t−1 and a(t+1) at time t+1, then a(t) is considered the peak.

(2) Threshold setting:

To ensure the validity of these peaks and valleys, the peak threshold is set. If the detected peak is less than the preset peak threshold, it will be discarded.

(3) Step determination:

If $T_{a\left(t\right)}-T_{a_{step}\left(i-1\right)}>T_{th}$, the peak is recorded as a valid peak. $T_{a_{step}\left(i-1\right)}$ is the time of the (*i* − 1)-th step, and $T_{th}$ is the training value of the time taken by the pedestrian to walking one step.

The step detection algorithm is depicted in Algorithm 1.
**Algorithm 1:** Step Detection Algorithm**Input**: Acceleration sequence a.**Output**: *i*-th step $a_{step}$.1: Setting Threshold $a_{th}$ and one-step taken time $T_{th}$;2: **for**
*i* = 1: a.length **do**3:  **if** a(t) is the Peak value **then**4:    **if**
$a\left(t\right)>a_{th}$ and $T_{a\left(t\right)}$-$T_{a_{step}\left(i-1\right)}>T_{th}$ **then**5:    $a_{step}\left(i\right)=a\left(t\right)$
6:    **end if**7:  **end if**8: **end for**

While walking, the step length in the current state is not only related to the current acceleration but also to the previous acceleration. It is not accurate to estimate the step length considering only the current motion state. 

Inspired by the literature [39], our method is not only related to the peak and valley values of acceleration in the current step but also accounts for the previous two steps. It can reflect the continuity and similarity of the moving process. Due to equipment errors during acceleration acquisition, we have added to compensate for the error caused by the equipment in the step length estimation. Meanwhile, during the estimation process, the attention mechanism scheme is also adaptively designed by calculating the difference between the maximum and minimum values of acceleration within each step, which has better adaptability than fixed weight estimation. 

The improved step length estimation method integrates the previous two step lengths as follows:

(23) $$l_{i}=c_{1}*l_{i-1}+c_{2}*l_{i-2}+c_{3}*K*\sqrt[{4}]{a_{i}^{peak}-a_{i}^{valley}}+\gamma$$

where $l_{i-1}$, $l_{i-2}$ are the (*i* − 1)-th and (*i* − 2)-th step length, respectively $\left[c_{1},c_{2},c_{3}\right]$ is the weighting vector, *K* is the model parameter, $a_{i}^{peak}$ and $a_{i}^{valley}$ are the peak and valley values of the acceleration in the *i*-th step, and γ is the accelerometer compensation, which is measured at a stationary time.

**Figure 6:**
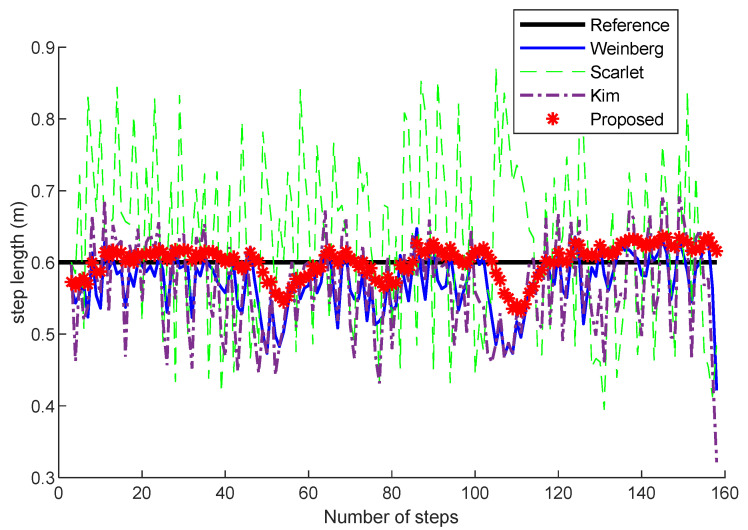
Comparisons of step length estimation at different step numbers with Scarlet, Kim, Weinberg and the proposed method.

Tests with several static states have been conducted in experiments. We have carried out some experiments with Scarlet [40], Kim [41], Weinberg [42] and our method. Figure 6 shows the comparison of the step length estimation at the trial site when the pedestrians walk at 0.6 m per step. The mean step length of our improved step model is 0.5939 m. The mean step lengths of the Weinberg, Scarlet, and Kim models are 0.5645 m, 0.6228 m, and 0.5567 m, respectively. The experimental results show that our method has higher stability and accuracy. This result is mainly due to the improved method, which can extract more features related to the current step than the Scarlet, Kim, and Weinberg methods. At the same time, the different attention values of each step are calculated adaptively and can predict more accurate pedestrian states.

- Heading direction estimation

According to Euler’s theorem, the quaternion-based attitude matrix description [43] is the transformation of the target coordinate system to the Earth coordinate system, as shown in Equation (24),

(24) $$C_{n}^{b}\left(q\right)=\left(\begin{array}{ccc} 2q_{0}^{2}-1+2q_{1}^{2} & 2\left(q_{1}q_{2}-q_{0}q_{3}\right) & 2\left(q_{1}q_{3}+q_{0}q_{2}\right)\\ 2\left(q_{1}q_{2}+q_{0}q_{3}\right) & 2q_{0}^{2}-1+2q_{2}^{2} & 2\left(q_{2}q_{3}-q_{0}q_{1}\right)\\ 2\left(q_{1}q_{3}-q_{0}q_{2}\right) & 2\left(q_{2}q_{3}+q_{0}q_{1}\right) & 2q_{0}^{2}-1+2q_{3}^{2} \end{array}\right)$$

where $C_{n}^{b}\left(q\right)$ denotes the rotation matrix of the quaternions. $q=\left(q_{1},q_{2},q_{3},q_{4}\right)$ denotes the posture quaternion, which is the unit vector, $q_{1}$ is the quaternion coefficient of the scalar part, whose value is equal to the cosine of half of the rotation angle of the coordinate system, $q_{2},q_{3},q_{4}$ denote the part of the vector, and $q_{1},q_{2},q_{3},q_{4}$ satisfied the following constraint:

(25) $$q_{1}{}^{2}+q_{2}{}^{2}+q_{3}{}^{2}+q_{4}{}^{2}=1$$

Since the gyroscope data can be used to estimate the heading angle from the differential equations of motion and gravitational acceleration and geomagnetic intensity can be used to correct the heading angle, the extended Kalman filter (EKF) is used to estimate the heading angle. The equation of state and the measurement equation of the EKF are expressed as Equations (26) and (27). The state vector is represented by the quaternion Q, and the measurement equation is a combination of the acceleration and magnetometer measurement data.

(26) $$Q_{k}=fQ_{k-1}+w_{k}$$

(27) $$\left[\begin{array}{c} acc_{k}\\ mag_{k} \end{array}\right]=\left[\begin{array}{cc} C_{n}^{b}\left(Q_{k}\right) & 0\\ 0 & C_{n}^{b}\left(Q_{k}\right) \end{array}\right]\left[\begin{array}{c} g\\ h \end{array}\right]+v_{k}$$

where $acc_{k}\;$and $mag_{k}$ measured by the acceleration and magnetometer sensors at time *k*, respectively, $f=e^{0.5\left(\vartheta \left(wt_{s}\right)\right)}$ is the state transfer matrix, $w_{k}$ is the process noise, and $v_{k}$ is the measurement noise. $C_{n}^{b}\left(Q_{k+1}\right)$ is the attitude rotation matrix, g is the normalized gravity vector, and h is the normalized magnetic field strength vector.

After the EKF prediction update, the optimal state vector can be iterated for each moment then the estimated optimal heading angle is as shown in Equation (28).

(28) $$a=arctan\left(\frac{2\left(q_{1}q_{2}+q_{0}q_{3}\right)}{2q_{0}^{2}-1+2q_{1}^{2}}\right)$$

- Dead reckoning

**Figure 7:**
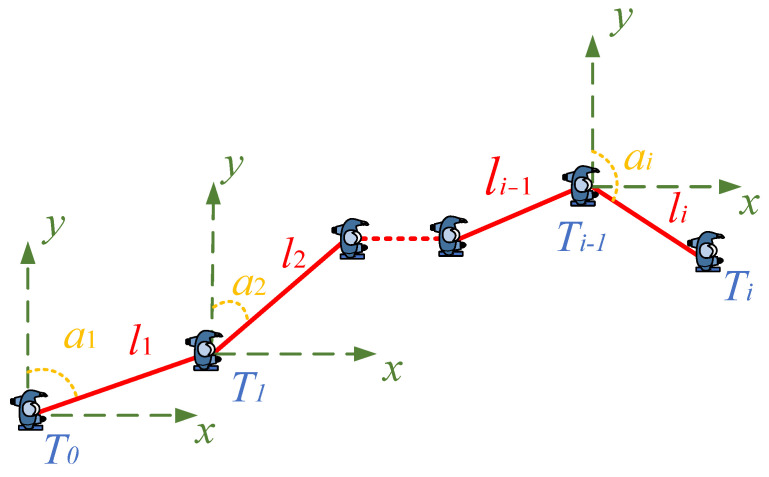
PDR motion model diagram.

The dead reckon schematic diagram (considering only the two-dimensional case) is shown in Figure 7.

Therefore, the current location $\left(x_{i},y_{i}\right)$ for the *i*-th step can be estimated according to the previous step, step length and heading direction. It is computed by the following formula:

(29) $$\{\begin{array}{c} x_{i}=x_{i-1}+l_{i}sina_{i}\\ y_{i}=y_{i-1}+l_{i}cosa_{i} \end{array}$$

where $l_{i}$ is the *i*-th step length and $a_{i}$ indicates the heading direction of the *i*-th step.

### 4.4. Adaptive Weight Value Generation

We propose an adaptive weight value generation scheme for acoustic signal estimation and IMU estimation. The weight value mechanism generates larger values for the important estimation and smaller values for the others. By generating the weight value, more generality and accuracy can be achieved.

Suppose at time *k*, given the location $m_{tk}=\left(x_{tk},y_{tk}\right)$ for TDOA estimation and $m_{pk}=\left(x_{pk},y_{pk}\right)$ for the IMU, at time *k* − 1, the location is $m_{k-1}=\left(x_{k-1},y_{k-1}\right)$.

For TDOA estimation, the distance for the current and previous times can be calculated as

(30) $$d_{t}=\|m_{k-1}-m_{tk}\|{}_{2}$$

For IMU estimation, the distance for the current and previous time can be calculated as

(31) $$d_{p}=\|m_{k-1}-m_{pk}\|{}_{2}$$

We can obtain $w_{tk}=\frac{1}{d_{t}}$, $w_{pk}=\frac{1}{d_{p}}$.

The normalized weights for time *k* can be obtained as

(32) $$w_{tk}=\frac{w_{tk}}{w_{tk}+w_{pk}}$$

(33) $$w_{pk}=\frac{w_{pk}}{w_{tk}+w_{pk}}$$

After normalization, the sum of the weight values is 1.

(34) $$w_{pk}+w_{tk}=1$$

### 4.5. Localization Estimation of the Fusion Method

In fusion localization, TDOA estimation is used to determine the initial location. To ensure accuracy, the optimal location is chosen by many trial tests.

Weight value generation can obtain the importance vectors. Formally, the weight value is defined as follows

(35) $$W_{t}=\left[w_{t1};w_{t2};\cdots ;w_{t\left(N-1\right)};w_{tN}\right]$$

(36) $$W_{p}=\left[w_{p1};w_{p2};\cdots ;w_{p\left(N-1\right)};w_{pN}\right]$$

where $w_{tk}$ and $w_{pk}$ are the estimated weight values of the TDOA and improved PDR methods, respectively.

Based on the estimation location from the two modules and the corresponding important values, we can estimate the current location $m_{k}=\left(x_{k},y_{k}\right)$ at time *k* as follows

(37) $$m_{k}=W\left[m_{t},m_{p}\right]$$

where *W* is the weight values.

However, due to the interferences of noise, multipath effects, and other factors, anomaly estimation is inevitable. During fusion localization, four cases occur. We propose setting the distance threshold $l_{th}$ scheme, which is adaptively adjusted according to the current and previous location. Experiments demonstrate that it is feasible to adopt 1.5 times the distance of the current location and estimation location. These cases are as follows:

*Case 1:* If the distance for the TDOA estimation is greater than the threshold: $d_{t}>l_{th}$, the estimation by the TDOA method may be an outlier and discarded. Then, the estimation of the PDR method is adopted for localization, that is, $m_{k}=m_{pk}$.

*Case 2:* If the distance for the PDR estimation is greater than the threshold: $d_{p}>l_{th}$, the estimation by the PDR method may be an outlier and discarded. Then, the estimation of the TDOA method is more accurate; therefore, $m_{k}=m_{tk}$.

*Case 3:* If the distance for the PDR and TDOA estimation is greater than the thresholds $d_{t}>l_{th}$ and $d_{p}>l_{th}$, the estimations are anomaly location. Return to estimate target.

*Case 4:* The distance for the PDR and TDOA estimation is less than the threshold: $d_{t}<l_{th}$ and $d_{p}<l_{th}$. Then, fusion localization will perform.

## 5. Illustrative Experimental Results

**Figure 8:**
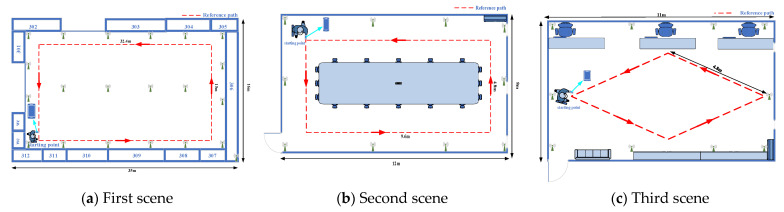
Floorplans of the experimental sites: (**a**) scene 1 with open spaces, (**b**) scene 2 includes small spaces with some tables and chairs, (**c**) scene 3 includes small spaces with some tables, chairs and cabinet.

To evaluate the performance of our proposed multimodal fusion system, we conducted extensive experiments at three different experimental sites, which include a 35 × 16 × 3 m^3^ area and a 12 × 8 × 3 m^3^ area and 11 × 9 × 3 m^3^ area. Figure 8 shows the floorplans of these trial sites. Covering 560 m^2^, the first scene includes open spaces. The second scene covers approximately 96 m^2^, is relatively small and includes some tables and chairs. The third scene covers approximately 99 m^2^, is relatively small and includes some tables, chairs, and cabinet. First, we detail the experimental setting in Section 5.1. Then, the discussion and analysis are provided in Section 5.2, and finally, we illustrate the localization results in Section 5.3.

### 5.1. Experimental Setting

We have developed the acoustic and inertial fuse localization system, which includes a client application and a server program. The client application was installed on Android devices with IMU sensors. The server program was installed on a 64-bit work station with a Windows operating system.

*Client application:* The client was developed using the Android operating system. The pedestrian carries the mobile phone with installed the client application to capture accelerometer, gyroscope, and directional angle readings as they walk. During the data collection process, it not only records the time stamp when the sensor readings are taken but also collects the signals from the anchor nodes placed in different locations around the experimental trial sites.

*Server program:* The localization program is implemented on the work station, which employed a Windows operating system with 64 bits. After receiving sufficient data, they will be sent to the server. The server program extracts the ultrasonic and inertial sequences separately. The coarse determination is conducted using the acoustic and inertial sequences. Then, the fusion localization estimation is achieved based on the adaptive weight value with coarse determination.

At the first experimental trial site, twenty anchor nodes for periodically transmitting acoustic signals were installed, and the mobile device was used as the localization target. At the second and third experimental trial sites, ten anchors were installed as acoustic sources. 

In these experiments, we invited five volunteers to collect acoustic and IMU data. During walking, the volunteers move along the designated route with the target smartphone in hand, and the client program automatically collected acoustic and sensor readings.

### 5.2. Discussion and Analysis

#### 5.2.1. Step Length Estimation

To validate the effect of step detection at different walking speeds, we ask a volunteer to hold smartphone and walk along the survey path at slow, normal and fast speed. During walking, the mobile device points in the head direction, and the client collects the sensor’s data automatically. For three cases, the volunteer has collected five trial data, respectively.

**Figure 9:**
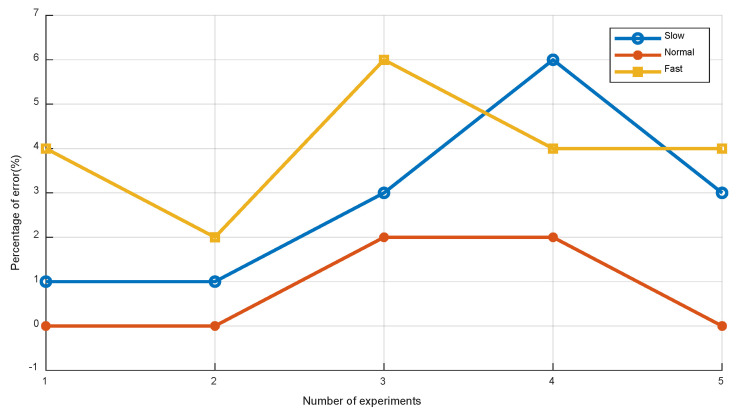
Step number error at different walking speeds.

Figure 9 shows the step number error at slow, normal, and fast speeds. It can be seen that the step detection can achieve better performance when the user walks at normal speed. This is due to the fact that the fluctuation of the acceleration is smaller and wave crests may be missed when the pedestrian walks at slow walking speeds. The fluctuation in the acceleration becomes greater at faster speeds, and the peaks and valleys may be misdetected, which results in an overchecked step count. Therefore, in our experiments, we let the pedestrians walk at a normal speed.

**Figure 10:**
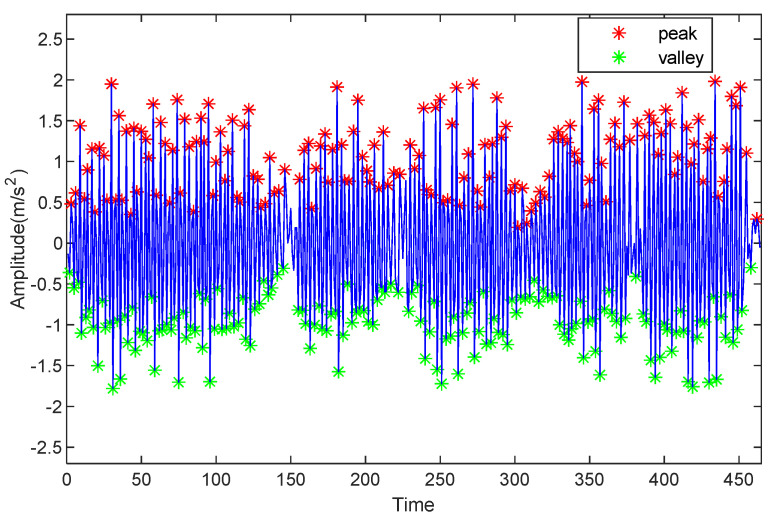
Peak and valley detection of acceleration.

In the step length estimation, the volunteer walks at normal speed and collects the IMU data. Figure 10 shows the peak detection results for the pedestrians in which the peaks and valleys of acceleration are labeled with red and green stars, respectively. This figure shows that all of the peaks and valleys in the acceleration are detected and can be achieved with high accuracy.

In this paper, we conducted experiments with the improved step length estimation at the trial site. Five volunteers are recruited and asked to collect data while walking along a corridor totaling 30 m with their cell phones in hand. Ten sets of IMU data are captured each time. Table 1 shows the solution distances and errors corresponding to each volunteer. The experimental results demonstrate that the improved step length method can achieve more accurate than the traditional algorithm, and the errors are not greater than 1.5%. The improved method has more pervasiveness.

#### 5.2.2. Improved PDR Estimation Results

**Figure 11:**
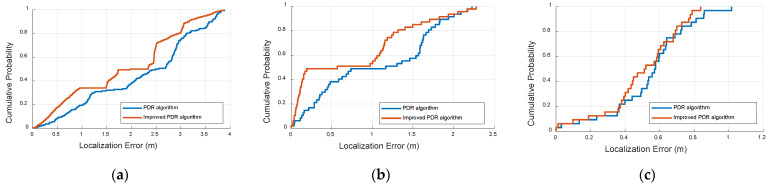
CDF of localization errors between the improved PDR and traditional PDR method at three sites. (**a**) First scene, (**b**) Second scene, (**c**) Third scene.

Figure 11 illustrates the cumulative density function (CDF) of the localization error for the traditional PDR method and improved PDR method in the three scenes, in which the abscissa is the localization error, the unit is meter, and the ordinate is the corresponding CDF. Figure 11 shows that the proposed method has a smaller localization error than the PDR method, which occurs due to the fact that our method can extract more features from the IMU data to improve the localization estimation.

### 5.3. Localization Performance

**Figure 12:**
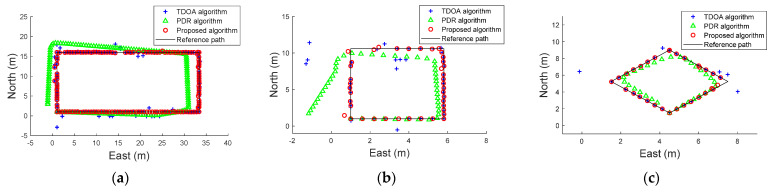
Localization track map with TDOA algorithm, PDR algorithm and proposed algorithm at three experimental sites. (**a**) First scene, (**b**) Second scene, (**c**) Third scene.

In this section, we conducted a localization experiment on the global region in the three scenes. The user walks along all of the planned paths in the whole scene to collect the IMU data and acoustic signals. Figure 12 shows the localization results using different algorithms. The TDOA estimation based acoustic signals can achieve good accuracy. However, some anomaly points occur. The errors in the PDR estimation increase over time, and the localization path increasingly deviates from the original path. The experiments indicate that the proposed method can achieve better localization estimation at the different planned paths than state-of-the-art algorithms. This is mainly due to the following two reasons. First, we introduce the threshold detection scheme into fusion localization, which can effectively suppress the anomaly points of TDOA outliers and the cumulative error of PDR estimation. In addition, the weight value generation can be of greater importance in TDOA and PDR estimations than the fixed weight value.

**Figure 13:**
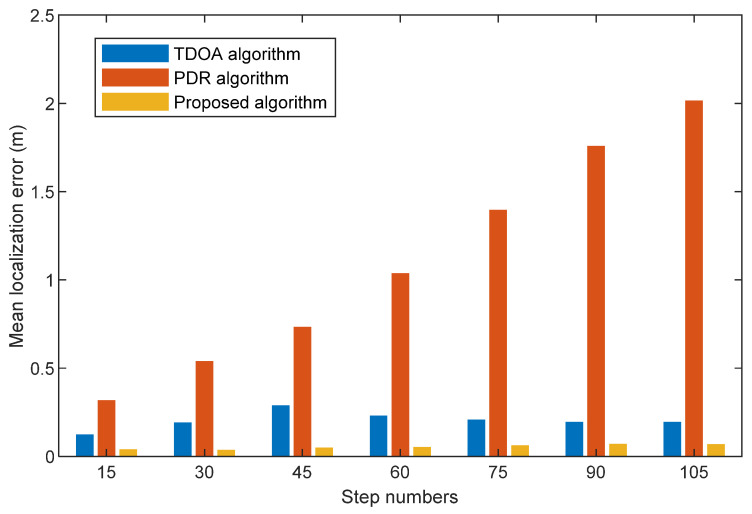
Mean localization error comparison with TDOA algorithm, PDR algorithm, and the proposed algorithm at different step numbers (First scene).

**Figure 14:**
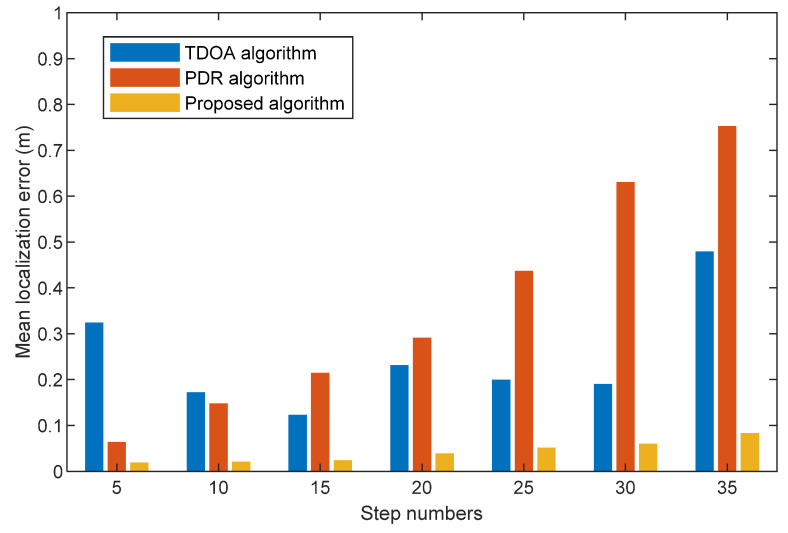
Mean localization error comparison with TDOA algorithm, PDR algorithm, and the proposed algorithm at different step numbers (Second scene).

**Figure 15:**
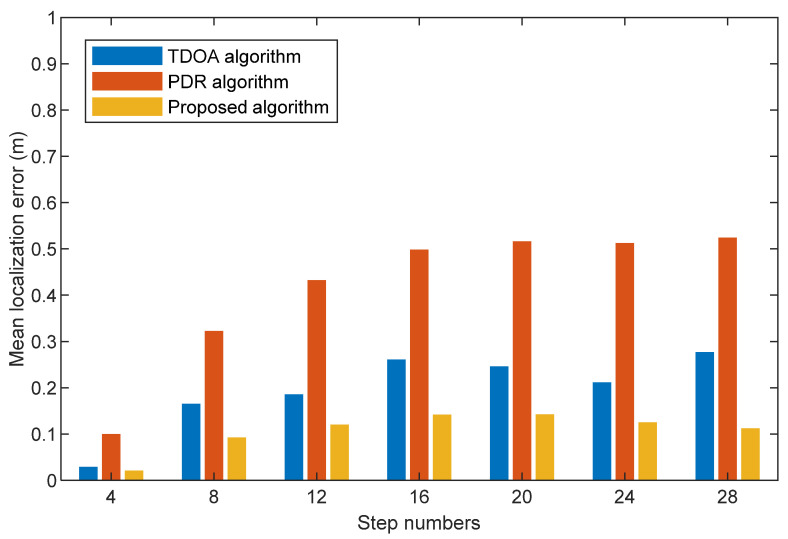
Mean localization error comparison with TDOA algorithm, PDR algorithm, and the proposed algorithm at different step numbers (Third scene).

We compare the mean localization errors among TDOA, PDR, and the proposed algorithm with different step numbers at three scenes in Figure 13, Figure 14 and Figure 15. They show that the mean localization errors for the TDOA method are generally stable. Specifically, the proposed algorithm does not vary significantly in localization accuracy with different travel paths. The mean localization for the PDR algorithm has an increasing localization error over time due to the cumulative error. Compared with the TDOA and PDR algorithms, our proposed fusion algorithm has the smallest mean localization error. This is due to the fact that our proposed threshold value based on step size removes the anomalies generated by acoustic signal localization, and acoustic signal localization corrects the problem of the cumulative errors generated by PDR localization.

**Figure 16:**
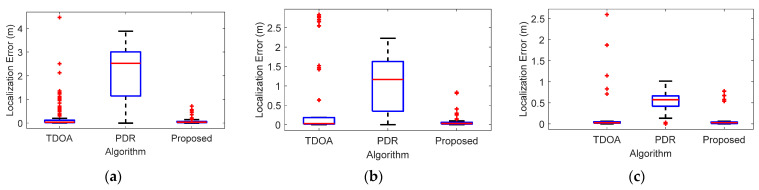
Localization error with TDOA algorithm, PDR algorithm and the proposed algorithm at different scenes. (**a**) First scene, (**b**) Second scene, (**c**) Third scene.

We present the localization errors incurred at the three trial sites in Figure 16. The experiment illustrates that the proposed method achieves comparable localization accuracy. The fusion scheme has an effective method to gain enough information. Consequently, it is able to detect the anomalies and cumulative errors caused by PDR localization.

**Figure 17:**
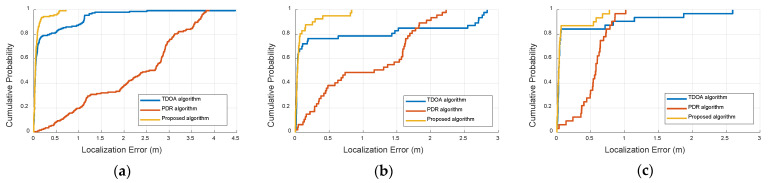
CDF of localization errors with TDOA algorithm, PDR algorithm and the proposed algorithm at different scenes. (**a**) First scene, (**b**) Second scene, (**c**) Third scene.

Figure 17 illustrates the cumulative density function (CDF) of the localization error in the three scenes, where the abscissa is the localization error, the unit of measurement is meters, and the ordinate is the corresponding CDF. From Figure 17a, it can be seen that the TDOA estimation is approximately 0.35 m when the accumulative probability accuracy is 80%. The PDR estimation is above 3.12 m when the accumulative probability accuracy is 80%. The proposed algorithm is approximately 0.08 m when the accumulative probability accuracy is 80%. It is therefore shown that the proposed method has better localization performance than TDOA and PDR estimation. From Figure 17b, it can be seen that the TDOA estimation is approximately 1.43 m when the accumulative probability accuracy is 80%, and the PDR estimation is above 1.69 m when the accumulative probability accuracy is 80%. The proposed algorithm is approximately 0.07 m when the accumulative probability accuracy is 80%. From Figure 17c, it can be seen that the TDOA estimation is approximately 0.06 m when the accumulative probability accuracy is 80%, and the PDR estimation is above 0.72 m when the accumulative probability accuracy is 80%. The proposed algorithm is approximately 0.05 m when the accumulative probability accuracy is 80%. These results show that the proposed fusion algorithm achieves sufficient accuracy compared with the state-of-the-art algorithms. This is due to the fact that the weight value generation can adaptively identify the importance between the TDOA estimation and PDR estimation. The threshold schemes can detect effective outliers.

Table 2, Table 3 and Table 4 demonstrate the mean error and root mean squared error (RMSE) of the different localization methods at the trial sites. In the first scene, the mean error and RMSE of our method are 0.0704 m and 0.139 m, respectively. In the second scene, the mean error and RMSE of our method are 0.094 m and 0.2066 m, respectively. In the third scene, the mean error and RMSE of our method are 0.1041 m and 0.2348 m, respectively. For the three sites, these values show that the localization performances in the proposed fusion localization have been greatly improved, which occurs since the proposed method can supply sufficient information about anomalies, effectively eliminate the errors, and guarantee the fluctuation of the localization estimation around the real value.

Table 5, Table 6 and Table 7 show the four percentile of error magnitude at 0.5, 0.75, 0.9, and 0.95 for the three trial sites. The experimental results illustrate that the proposed method has better localization performance at different walking paths in these indoor scenes than the state-of-the-art methods. This is due to the fact that the fusion scheme is able to provide effective information for localization.

## 6. Conclusions

In this paper, to solve the problem of anomalies in acoustic signal localization, we propose a step threshold method that can set the threshold value based on the step length of the previous step and thus solve the anomalies in localization. Then, in the PDR localization system, we improve the traditional step estimation algorithm model and propose an improved model that adaptively estimates the current step length based on the previous two-step length and the current acceleration value. Finally, we fuse acoustic estimation and improved PDR estimation to achieve the pedestrian’s location by generating distance weights. An indoor localization system by fusing the TDOA and improved PDR estimation is achieved high localization performance and low cost. We have conducted extensive experiments at three sites. The experimental results demonstrate that the average localization error of our method achieves higher accuracy compared with the state-of-the-art algorithms. Considering the compatibility of mobile phones with ultrasonic and inertial signals, our localization system has a relatively low cost.

## Figures and Tables

**Table 1:** Comparison results between the Weinberg method and proposed step length estimation method (m).

Number	Weinberg Method		Proposed Method	
	Distance Estimation	Absolute Error	Distance Estimation	Absolute Error
1	28.1776	1.8224	29.6361	0.3639
2	28.2316	1.7684	29.6584	0.3416
3	28.4092	1.5908	29.7899	0.2101
4	28.6329	1.3671	30.1634	0.1634
5	28.3142	1.6858	29.8298	0.1702

**Table 2:** Comparison of localization accuracy with different algorithms (m) (First scene).

Method	Mean Error	RMS Error
TDOA	0.2587	0.6048
PDR	2.2097	2.4705
Improved PDR	1.8363	2.1433
Proposed algorithm	0.0704	0.1390

**Table 3:** Comparison of localization accuracy with different algorithms (m) (Second scene).

Method	Mean Error	RMS Error
TDOA	0.5396	1.1167
PDR	1.0320	1.2544
Improved PDR	0.7431	1.0253
Proposed algorithm	0.0940	0.2066

**Table 4:** Comparison of localization accuracy with different algorithms (m) (Third scene).

Method	Mean Error	RMS Error
TDOA	0.2467	0.6318
PDR	0.5431	0.5882
Improved PDR	0.4981	0.5424
Proposed algorithm	0.1041	0.2348

**Table 5:** Comparison of quantiles of error with different algorithms (m) (First scene).

Method	50th Percentile	75th Percentile	90th Percentile	95th Percentile
TDOA	0.0375	0.1225	1.0941	1.1265
PDR	2.5225	3.0064	3.6152	3.7256
Improved PDR	2.1554	2.6888	3.1604	3.5094
Proposed algorithm	0.0349	0.0702	0.1365	0.3713

**Table 6:** Comparison of quantiles of error with different algorithms (m) (Second scene).

Method	50th Percentile	75th Percentile	90th Percentile	95th Percentile
TDOA	0.0296	0.1842	2.6952	2.7638
PDR	1.1646	1.6289	1.9164	2.1028
Improved PDR	0.5679	1.2240	1.7996	2.0691
Proposed algorithm	0.0320	0.0617	0.2682	0.5881

**Table 7:** Comparison of quantiles of error with different algorithms (m) (Third scene).

Method	50th Percentile	75th Percentile	90th Percentile	95th Percentile
TDOA	0.0264	0.0493	0.9246	1.7991
PDR	0.5731	0.6645	0.8243	0.8578
Improved PDR	0.5154	0.6784	0.7711	0.7867
Proposed algorithm	0.0278	0.0445	0.5559	0.6693

## Data Availability

All test data mentioned in this paper will be made available on request to the corresponding author’s email with appropriate justification.

## Notations

$t_{A_{i}}^{s}$	The time when anchor *A_i_* transmits a message
$t_{A_{i}}^{rj}$	The time when anchor *A_j_* receive the message from anchor *A_i_*
Δt	The transmission interval between anchors
$d_{A_{i}A_{j}}$	The distance from anchor *A_i_*’s speaker to anchor *A_j_*’s microphone
$d_{ij}$	The distance difference from anchors *A_i_* and *A_j_* on Target
$d_{i}$	The distance between the target *M* and anchor *A_i_*
$q_{1}$	The scalar part of the quaternion coefficients
$q_{2},q_{3},q_{4}$	The vector part of the quaternion coefficient
$Q_{k}$	The state vector at time *k*
$acc_{k}$	The acceleration measured by the accelerometer at time *k*
$mag_{k}$	The data measured by the magnetometer sensor at time *k*
$m_{k}$	The location estimation at time *k*
$m_{tk}$	The location estimation using TDOA method at time *k*
$m_{pk}$	The location estimation using PDR method at time *k*
$d_{t}$	The distance between the current and previous time using TDOA method
$d_{p}$	The distance between the current and previous time using PDR method
$w_{tk}$	Weight value in TDOA method at time *k*
$w_{pk}$	Weight value in PDR method at time *k*
$W_{t}$	Weight value in TDOA method
$W_{p}$	Weight value in TDOA method
$l_{th}$	Distance threshold
